# Memory properties and charge effect study in Si nanocrystals by scanning capacitance microscopy and spectroscopy

**DOI:** 10.1186/1556-276X-6-163

**Published:** 2011-02-22

**Authors:** Zhen Lin, Georges Bremond, Franck Bassani

**Affiliations:** 1 Institut des Nanotechnologies de Lyon, UMR 5270, Institut National des Sciences Appliquées de Lyon, Université de Lyon, Bât. Blaise Pascal, 20, avenue Albert Einstein - 69621 Villeurbanne Cedex, France; 2 Institut Matériaux Microélectronique Nanosciences de Provence, UMR CNRS 6242, Avenue Escadrille Normandie-Niemen-Case 142, F-13397 Marseille Cedex 20, France

## Abstract

In this letter, isolated Si nanocrystal has been formed by dewetting process with a thin silicon dioxide layer on top. Scanning capacitance microscopy and spectroscopy were used to study the memory properties and charge effect in the Si nanocrystal in ambient temperature. The retention time of trapped charges injected by different direct current (DC) bias were evaluated and compared. By ramp process, strong hysteresis window was observed. The DC spectra curve shift direction and distance was observed differently for quantitative measurements. Holes or electrons can be separately injected into these Si-ncs and the capacitance changes caused by these trapped charges can be easily detected by scanning capacitance microscopy/spectroscopy at the nanometer scale. This study is very useful for nanocrystal charge trap memory application.

## 

Recently, the self-assembled silicon nanocrystals (Si-ncs) that are formed within ultrathin SiO_2 _layer are considered to be a promising replacement of this conventional floating gate [1,2]. These isolated Si-ncs embedded in between a tunnel and a top dielectric layer serve as the charge storage nodes and exhibit many physical properties even at room temperature such as Coulomb blockade [3], single-electron transfer [4] and quantization charges effect [5] which differ from bulk crystals. It can reduce the problem of charge loss encountered in conventional memories, cause thinner injection oxides and hence smaller operating voltages, better endurance and faster write/erase speeds. So, the characterisation and understanding of its charging mechanism in such nanostructure is of prime importance.

Although the conventional I-V and C-V characterization methods for memory application provide a vast amount of macro information, these methods lack the ability of discriminating structural and material properties on a nanometer scale. Since atomic force microscopy (AFM) was invented by Binning and Rohrer in IBM, 1982 (Nobel Prize awards in 1986), it has become a powerful high-spatial-resolution tool for nanoscale semiconductor analysis or characterization comparing to several conventional methods for such as x-ray, nuclear, electron and ion beam, optical and infrared and chemical technique. It can provide simultaneous topography and various physical feature images with some additional electrical applications such as scanning capacitance microscopy (SCM) [6,7], electrostatic force microscopy (EFM) [8], scanning resistance microscopy [9] and Kelvin probe force microscopy [10]. In amount of these techniques, SCM became one of the most useful methods for the capacitance characterization of semiconductor as its non-destructive detection of varies electrical properties with high resolution such as dopant profiling variation [11], silicon p-n junction [12] and carrier injection [13], etc.

In this letter, scanning capacitance microscopy and spectroscopy (SCS) were used to study the memory properties and charge effect of the Si-ncs materials in ambient temperature.

Figure 1 shows the formation of these isolated Si-ncs. First, a 4-nm-thick thermal oxide was grown as the tunnelling oxide on an amorphous Si substrate. Subsequently, Si layer was deposited by molecular beam epitaxy over a very thin SiO_2 _layer, 5 nm in thickness, at ambient temperature and was thermally annealed at 750°C for 20 min under ultrahigh vacuum. The dewetting process leads to the formation of isolated Si nanocrystals having an average density of 4 × 10^10 ^cm^-2^.

**Figure 1:**
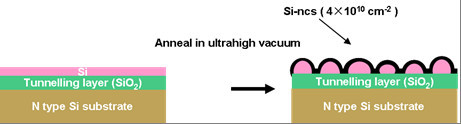
**The formation of isolated Si-ncs**.

Veeco Digital Instruments 3100 Dimensions AFM employing a Nanoscope V controller was used to conduct SCM and SCS measurements. The conductive tip that was selected was commercial Arrow-EFM PtIr coating tip. It has an average tip radius of less than 10 nm, cantilever spring constant: 2.8 N/m and resonance frequency: 75 kHz. SCM images were taken with a fixed bias frequency of 50 kHz, SCM lock-in phase of 90°and capacitance sensor frequency of 910 MHz. The amplitude of direct current (DC)/direct voltage signal is strongly dependent on the modulation voltages and the magnitude of capacitance variation is generally a nonlinear function of the carrier concentration. Figure 2 shows the topography and SCM image. The contrast between Si-nc and the oxide layer was clear in SCM image which indicates that the Si-nc has different capacitance from the oxide layer.

**Figure 2:**
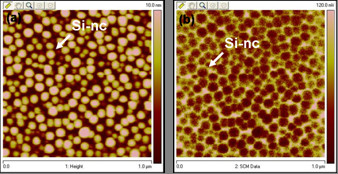
**Si nanocrystal images**. **(a) **Topography **(b) **SCM data image.

In order to investigate the effects of DC bias and alternating current (AC) bias to the SCM signal, the slowscan was disabled and a typical line scan was performed. In Figure 3a, the VAC bias was fixed to 2,500 mV. The SCM image and signal variation with DC bias is shown in Figure 3a. Different DC bias during the scan can cause different SCM signal. The best signal/noise ratio and highest contrast occurred when -1 or 0.5 V was applied, which is the same as Ge nanocrystals. Too high DC bias amplitude, such as up to 2 V, will make the SCM signal disappeared. Figure 3b illustrates this variation in function to the DC bias. The higher the DC bias amplitude, the stronger SCM signal intensity was. However, the lower the contrast between the Si-ncs and dielectric layer was. Positive or negative modulation corresponds to different SCM phase. The best resolution and the best signal to noise ratio which correspond to the highest contrast between Si-ncs and dielectric layer in the image was obtained near -0.5 and 0.5 V with the scan rate of 0.5 Hz.

**Figure 3:**
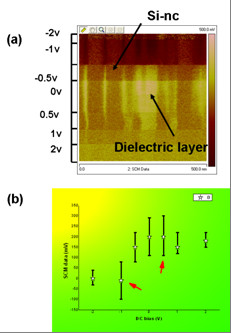
**SCM image (a) and signal (b) versus different DC bias**.

AC bias was also investigated by fixing the DC bias to 0.5 V which is one of the best DC bias as we mentioned above. The SCM line scan image with different AC bias and its variation was shown in Figure 4. The contrast between the Si-ncs and dielectric layer changed with AC bias. The higher the AC bias, the stronger the SCM signal intensity was. However, too high AC voltage can induce charge injection in the sample which will create parasitic capacitance and high noise. Here, 2 V AC bias was fixed during the scan.

**Figure 4:**
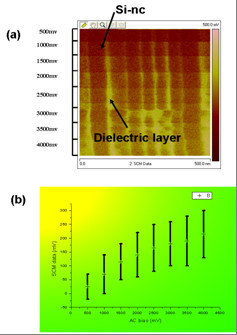
**SCM image (a) and signal (b) versus different AC bias**.

Charge injection was done by separately applying (0.5, 1.0, 2.0, and 3.0 V) to the tip during the contact SCM scan. Then DC bias was set back to -0.5 V which was the best as we chose for our signal. As the SCM signal is dependent on the quantity of injected charges, it was monitored for charge retention time study. The non-linear function between the retention time and the DC bias is shown in Figure 5. The higher the DC bias (charging voltage) was, the longer its discharge time was, which means more carriers were injected into the Si-nc. Holes are much easier to be injected than electrons as the retention time of positive charging was longer than the negative charging with respect to the same DC charging intensity. When charge injection was done by more than 7 V, the charging process can't be detected in several minutes. This indicates that the charges were trapped by the Si-nc which made the retention time much longer.

**Figure 5:**
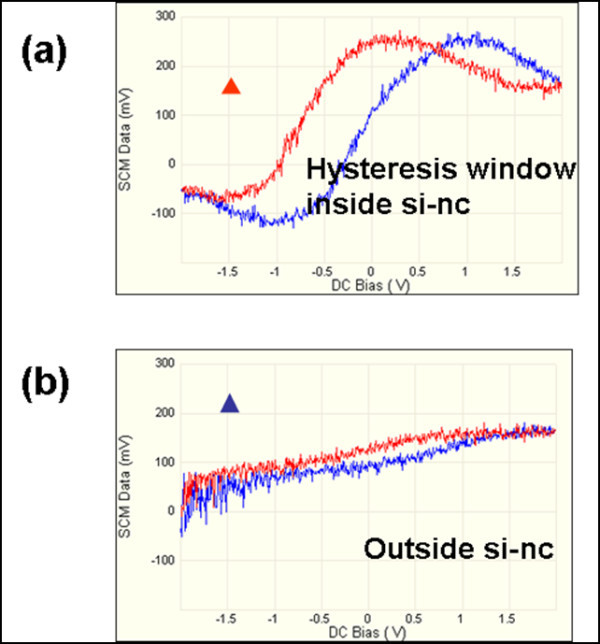
**Charge and discharge with different DC bias**.

Ramp processes between -2 and +2 V were done by SCS separately on and outside an isolated Si-nc without charge injection. Strong hysteresis window was observed on the isolated Si-nc. But outside the dot, this effect was too weak (see in Figure 6). Furthermore, SCS was used to quantitatively investigate trapped charge effect inside the isolated Si-nc. From the SCS signals, the curve shift direction and distance were observed differently by applying a DC bias of -10 or +10 V to the tip during charging (see in Figure 7). There is a shift of 0.91 V by +10-V charge while -0.74 V shift by -10-V charge. This relates to the fact that different type of carriers can be injected into these Si-ncs and the capacitance changes caused by these trapped charges can be easily detected by SCM at the nanometer scale. It also verified the previous conclusion that holes are much easier to be injected and trapped than electrons.

**Figure 6:**
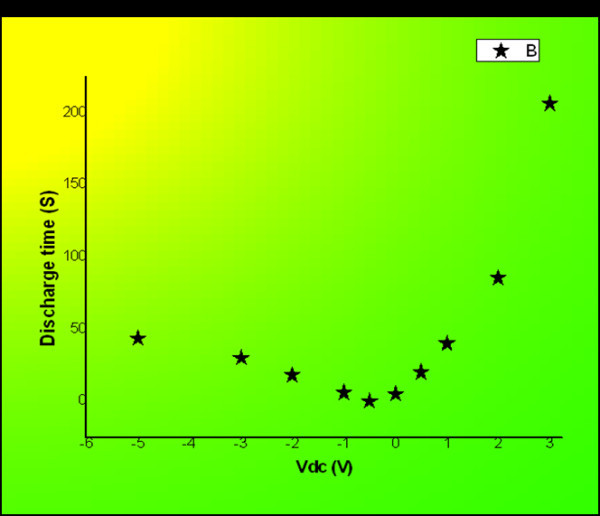
**Ramp process for hysteresis window by SCS**.

**Figure 7:**
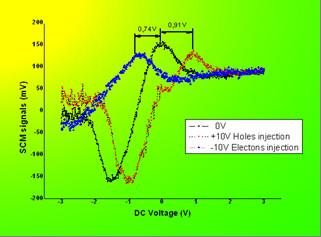
**SCS curve shift after charge injection by +10 and -10 V**.

In this letter, Si-ncs were formed on top of a thermally grown silicon dioxide layer. SCM and SCS were used to study the memory properties and charge effect on the Si-ncs in ambient temperature. Applying DC bias to the conductive tip, charges were injected into the Si-ncs which was recorded by the SCM images. The retention time of these trapped charges injected by different DC bias were evaluated and compared. By ramp process, strong hysteresis window was observed from the SCS signal. Furthermore, the SCS curve shift direction and distance were observed differently for quantitative measurements. This relates to the fact that holes or electrons can be separately injected into these Si-ncs and the capacitance changes caused by these trapped charges could be easily detected by SCM/SCS at the nanometer scale.

## Authors' contributions

ZL carried out the SCM and SCS experiment, studied these results and drafted the manuscript. GB participate the study of experiment results and manuscript writing. FB conducted the sample fabrication and the discussion. All authors read and approved the final manuscript

## Competing interests

The authors declare that they have no competing interests.
